# Ablation of Left Atrial Tachycardia following Catheter Ablation of Atrial Fibrillation: 12-Month Success Rates

**DOI:** 10.3390/jcm11041047

**Published:** 2022-02-17

**Authors:** Armin Luik, Kerstin Schmidt, Annika Haas, Laura Unger, Panagiotis Tzamalis, Bernd Brüggenjürgen

**Affiliations:** 1 Karlsruhe Municipal Hospital, Academic Teaching Hospital of the University of Freiburg, 76133 Karlsruhe, Germany; kerstin.schmidt@klinikum-karlsruhe.de (K.S.); annika.haas@klinikum-karlsruhe.de (A.H.); panagiotis.tzamalis@klinikum-karlsruhe.de (P.T.); 2 Institute of Biomedical Engineering, Karlsruhe Institute of Technology, 76131 Karlsruhe, Germany; laura.unger@kit.edu; 3 Institute for Health Services Research and Technical Orthopaedics, Hanover Medical School, 30625 Hannover, Germany; bernd.brueggenjuergen@t-online.de

**Keywords:** atrial tachycardia, atrial flutter, catheter ablation, critical isthmus, long term success

## Abstract

The treatment of atrial tachycardia following catheter ablation of atrial fibrillation is often challenging. Electrophysiological studies using high-resolution 3D mapping systems have contributed significantly to their understanding, and new ablation approaches have shown high rates of acute terminations with low recurrences for the clinical AT. However, patient populations are very heterogeneous, and long-term data of the freedom from any atrial tachycardia or any arrhythmia are still sparse. To evaluate long-term success, a unified patient population and predefined ablation strategies are preferred. In this study, we present 12-month success and mean 30 month follow-up data of catheter ablation of left atrial tachycardia. All 35 patients had a history of pulmonary vein isolation (PVI), 71% of which had a previous substrate modification. A total of 54 ATs, with a mean cycle length 297 ± 86 ms, 31 macro-reentries, and 4 localized reentries, were targeted. The ablation strategy to be used was given by the study protocol, depending on the type of reentry and the number of critical isthmuses. All available ablation strategies were included: standard (anatomical) lines, individual lines, critical isthmuses, and focal ablation. All ATs were terminated by ablation. A total of 91% terminated upon the first ablation strategy. Freedom from any AT after 12 months was 82%, and from any arrhythmia, it was 77%. The multi-procedure success after 30 months was 65% for any AT and 55% for any arrhythmia. In conclusion, individual ablation strategies based on the reentry mechanism and the number of critical isthmuses seems promising and demonstrates a high long-term clinical success. Tachycardia comprising a single critical isthmus can be ablated by critical isthmus ablation only. These patients present with the highest 12-month and long-term success rates.

## 1. Introduction

The crucial mechanism of atrial tachycardia (AT) can be very diverse. The incidence is age- and gender-dependent, with 5/100,000 in patients under 50 years and approximately 600/100,000 in subjects >80 years of age [1,2]. Left atrial tachycardia most commonly occurs after previous cardiac surgery or catheter ablation of atrial fibrillation (AF), but it can also be idiopathic. The reported prevalence of AT after AF catheter ablation (CA) ranges from 2% to 20% [3,4,5] and is mostly associated with structural heart disease [3]. Although external cardioversion (CV) and medical rate control are recommended by the recent ACC/AHA/HRS guidelines for acute treatment, relapse rates after CV are high [6]. CA offers a promising, however, challenging and time-consuming, alternative. Reported acute success rates of CA, mostly defined as restoring the sinus rhythm (SR) rather than non-inducibility of arrhythmias, vary between 73% and 93% [6,7,8,9,10,11,12,13,14,15]. Reported recurrence rates for AT are clinically relevant and vary considerably between 7% and 53% [6,8,10,11,12,13,14,15,16] with one study reporting an average time to recurrence of 49 days. Focal firing, spiral excitation patterns, and reentry were found to be driving mechanisms of ATs. Reentry, mainly named as atrial flutter, is the most frequently observed mechanism and can be subdivided into macro- and micro-reentry mechanisms [17,18]. The complexity and variability of these circuits is related to the presence of zones of block, slow conduction, and electrically silent areas. 3D electro-anatomical mapping systems have improved the understanding of complex AT mechanisms while facilitating mapping as well as guiding ablation [19,20,21]. However, depending on the alterations of the underlying substrate, especially the presence of areas of a very low voltage, the interpretation of the AT may be misleading, even by the use of high-resolution mapping systems. Stimulation maneuvers, on the other hand, may alter the AT into another arrhythmia [22,23]. Macro-reentry tachycardias were classified most commonly as roof-dependent or peri-mitral and treated by an anatomical approach. Takigawa et al. introduced the practical isthmus, usually the narrowest bridge of conductive tissue between scars or anatomic obstacles, as the easiest site for ablation [24]. Another possible ablation target is the slow conducting area in the mid-diastole of the excitation, often referred to as the critical isthmus [25,26]. 

In this type of arrhythmia, the patient cohort is usually very heterogeneous. This study wanted to create comparable outcomes by applying predefined ablation strategies, depending on the type of reentry and the number of critical isthmuses. The primary objectives were the 12-month success and mean long-term success rates. 

## 2. Materials and Methods

### 2.1. Study Design

**Figure 1:**
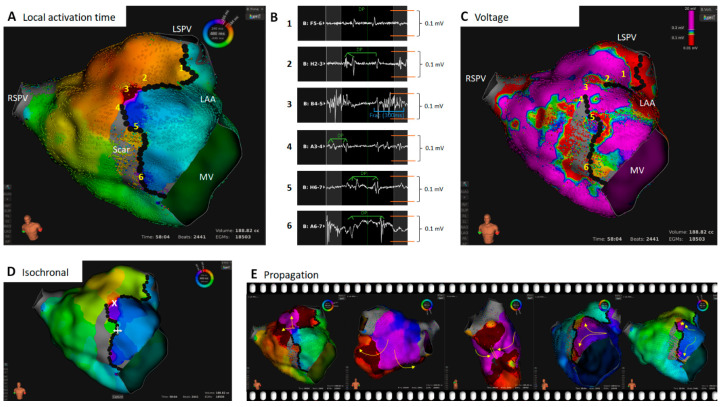
Patient with a persistent AF and a history of previous RF isolation of the PVs. The cycle length of the AT was 480 ms. Shown is the ultra high-resolution map with the Rhythmia HDx™ 3D mapping system. (**A**): Local activation time map of the left atrium. Black dots represent locations with double potentials (DP). (**B**): Local bipolar electrograms. The numbers correspond to the locations in the LAT (**A**) and voltage map (**C**). (**C**): Voltage map; signal amplitude (sa) > 0.3 mV = violet, sa < 0.1 mV = red, 0.1 mV > sa < 0.3 mV = rainbow color, sa < 0.015 mV = grey (noise level). Black dots represent locations with double potentials (DP). (**D**): LAT Isochronal, step size 30 ms; critical isthmus marked with a white cross. (**E**): Propagation maps. Summary: The patient presented a macro-reentry AT with one critical isthmus. The dominant reentry propagated across the roof around the left PVs (**A**,**E**). A large scar area at the anterior wall was striking (**C**). The isochronal map showed slow conduction at the upper and lower end of the scar area (**D**). Based on the voltage map (**C**), it was not feasible to delineate the distinct borders of the slow conducting area. A review of the electrograms revealed double potentials in areas 1 and 2 (**B**). However, another breakthrough at the lower end of the low voltage area appeared to be possible. Again, an electrogram review revealed double potentials in areas 4–6 (**B**). Of note: all signals which had two peaks within one AT cycle and were separated by an isoelectric line were classified as double potentials. This was also true if the single peaks were very close together or fractionated. A highly fractionated electrogram was found in area 3. Therefore, this spot was defined as a critical isthmus. The ablation strategy was a critical isthmus ablation only, which terminated the AT during the first RF delivery. Abbreviations: LSPV = left superior pulmonary vein; RSPV = right superior pulmonary vein; MV = mitral valve; LAA = left atrial appendage; DP = double potential.

This study was designed as a prospective, single group study. Inclusion criteria were sustaining left AT in patients with a history of AF and prior pulmonary vein isolation (PVI). Exclusion criteria were (1) an age younger than 18 years and (2) any contraindication to CA. The patients were treated with a substrate-based individualized ablation protocol (Figure 1) and followed up every six months. The study was conducted in accordance with the provisions of the Declaration of Helsinki and was approved by the ethics committee of the University of Stuttgart, Germany. The electrophysiological study and mapping was performed with the Rhythmia HDx™ Mapping System (Version 4.0.1.3). The electrophysiological (EP) study was conducted in accordance with the current guidelines [27]. ATs were sequentially mapped with the IntellaMap Orion^TM^ multipolar basket catheter and Rhythmia HDx™ Mapping System. Local activation time (LAT) and voltage maps were acquired for all ATs. Electrogram annotation was performed automatically by the mapping system. After the map was completed, all efforts were redirected to track the propagating wavefront based on a local electrogram analysis, using the system’s virtual roving probe, and to gain a detailed understanding of the reentry circuit [28,29,30]. All interpretations were based on the electrograms recorded with the IntellaMap Orion™ catheter. If the cycle length was fully covered, a reentry mechanism was assumed. Based on the discussed AT mechanisms at the time of the study initiation, an AT was predefined as (i) macro-reentry if the dominant path looped around an anatomical obstacle. Reentries which did not incorporate an anatomical obstacle were classified as (ii) localized reentries and were further differentiated into small-area reentries or micro-reentries. A (ii,a) small-area reentry was defined as a propagation around a dense scar area or a line with double potentials, not incorporating an anatomical obstacle. For (ii,b) a micro-reentry, the encircled area is reduced to a single spot. Centrifugal activation from a distinct focal source covering only part of the cycle length was considered as (iii) a focal AT [28,29,31]. Stable, spiral activations were predefined as (iv) rotor [32].

### 2.2. Catheter Ablation Approaches

**Figure 2:**
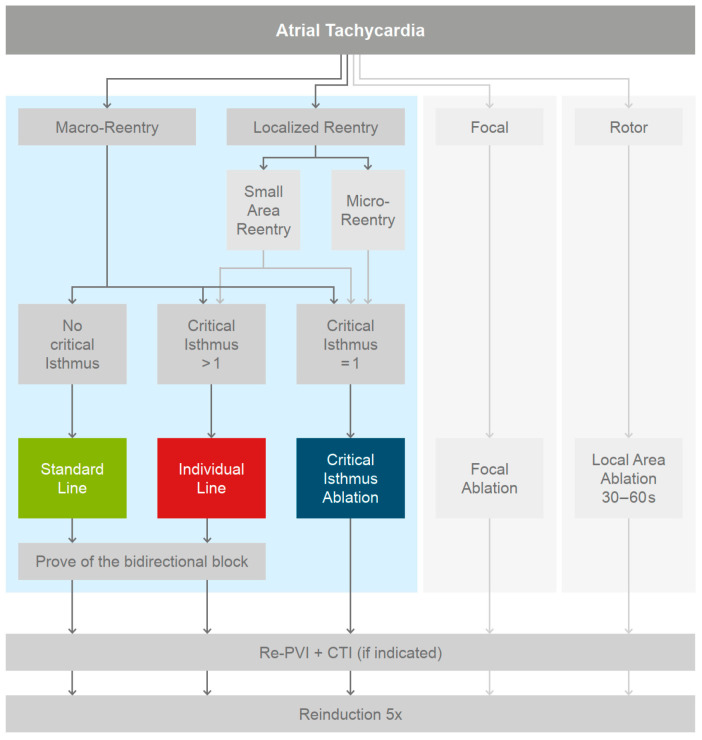
PVI = pulmonary vein isolation; CTI = cavotricuspid isthmus. Macro-reentry = circular activation around an anatomical obstacle. Localized reentry = circular reentry not involving an anatomical structure but around a spot presenting double potential (micro-reentry) or around an area or line of block (small-area reentry). Focal = true focal automatisms not related to structurally altered tissue, with a centrifugal excitation covering only part of the cycle length. Rotor = spiral excitation wavefronts.

Reentries were ranked by the size of the pathway, indicating its stability. Micro-reentries, focal sources, or rotors underwent a manual EGM analysis to rule out visualization artefacts. If the driving mechanism was found to be reentry, the propagating wavefront was further analyzed for isthmus areas (Figure 1). Such narrow, slow conducting bridges are always bordered by adjacent scar areas, lines of block, or anatomical structures, electrocardiographically mainly represented by double potentials. Within these areas, the slowest conduction presenting the highest fractionation of the electrogram was defined as the critical isthmus. The primary aim of the ablation was to restore SR with a minimum of ablated tissue. The primary ablation strategy was to target the AT first, even if PV reconnection was discovered, to prove the hypothesis of the underlying mechanism. The distribution of the ablation strategies is outlined in Figure 2. For macro-reentries, areas with a reduced conduction velocity, fractionation, and double potentials were highlighted within the dominant circuit. The critical isthmus was defined as described above. If only one critical isthmus was present, a critical isthmus ablation was the sole ablation strategy. In cases with more than one critical isthmus, the ablation was extended to an individual line, equivalent to the Takigawa approach. The individual line aimed to connect the critical isthmus, via areas of slow conduction, to a line while ablating as little healthy tissue as possible and finally anchoring each end to an anatomical structure. If no slow conducting areas were found within the dominant circuit, a standard line was performed. A roof line, a mitral isthmus line [33], and a modified anterior line [34,35] were considered suitable standard lines. A proof of bidirectional block was mandatory for all lines. Localized reentries were treated either with critical isthmus ablation or an individual line, according to the protocol (Figure 2). The voltage map was only used supportively in theory-building. In the case of small-area reentries presenting with two or more critical isthmuses, it was left to the physician’s discretion to choose the first ablation target. Subsequently, all additional critical isthmuses were ablated. Micro-reentries were locally ablated at their critical isthmus, including an additional ablation of fractionated electrograms in the immediate vicinity. Focal tachycardias were ablated at the site of the earliest bipolar and unipolar activation. The strategy for rotors was to ablate a slightly larger, local area at the earliest activated area for at least 30–60 s.

### 2.3. Further Ablation Strategies and Reinduction

After completion of the primary ablation strategy, all PV gaps were closed. Additionally, the cavotricuspid isthmus was ablated if common-type atrial flutter was suspected in the medical history. Repeated decremental atrial pacing of <200 ms was performed five times to test for non-inducibility. All inducible sustaining ATs were mapped and ablated using the same approach. If AF was induced, external cardioversion was performed. Ablation was performed using the Directsense™ technology (Boston Scientific, Charlestown, MA, USA) [33,36] in conjunction with the HAT 500 RF-generator (Osypka, Rheinfelden, Germany).

### 2.4. Study Endpoints and Follow-Up

The primary endpoint was clinical success after 12 months and long-term success. Secondary endpoints comprised (1) termination into SR overall, (2) termination into SR upon the primary ablation strategy as a proof of concept that the AT mechanism was completely decrypted, (3) time to termination in SR, and (4) the non-inducibility of any AT/AF. Follow-up was performed every 6 months in our outpatient clinic, including a medical history, 12-lead ECG, and 24 h Holter ECG, as well as a detailed interrogation for symptoms indicating possible AF or AT recurrence. In the case of a normal Holter ECG but reported arrhythmia episodes, patients received 7-day Holter-ECG monitoring to detect short arrhythmia episodes. The study did not include a blanking period. In the case of the recurrence of an incessant AT, a redo procedure was scheduled as soon as possible. Single-procedure and multi-procedure success was defined as the freedom from any AT after the index and the last ablation procedure, respectively.

### 2.5. Statistical Analysis

Variables are displayed as means with standard deviation. A Student’s *t*-test was used for the comparison of patients. Freedom from any arrhythmia was quantified by the Kaplan–Meier estimator. A *p*-Value of <0.05 was considered statistically significant. Analyses were performed using Excel Version 2201, Matlab 2021b, or R Version 3.3.2. 

## 3. Results

### 3.1. Baseline Characteristics

A total of 35 cases of incessant AT were included in the study. According to the inclusion criteria, all patients had a history of AF and had undergone previous PVI. The baseline characteristics are shown in Table 1. On average, the patients had 2 ± 1 pre-ablations in the left atrium, and the mean time to last left atrial ablation was 18 ± 23 months. With respect to all pre-procedures, 10 (29%) had a PVI only, and 25 (71%) had a previous PVI with additional substrate modification of the left atrium. A total of 13 (37%) had, in addition, previous ablation due to a left AT. In the PVI-only group, 36% were pre-ablated with cryo energy. A total of 43% had undergone CTI ablation. 

### 3.2. Investigated Mechanisms of the Tachycardia

A total of 54 tachycardias were mapped and ablated, with a maximum of four maps per patient. For the detailed analysis and calculations, only the map of the clinical AT was considered. Macro-reentries were present in 86% of the patients. Out of these, 42% (18/31) involved the mitral annulus, and 58% involved the left atrial roof. Reentries encircling the ipsilateral PVs of each side were counted as roof-dependent. A total of 14% presented with a localized reentry. Of the localized reentries, one was classified as a small-area reentry and three as micro-reentries. Focal activations and rotors were not found in this study. The dominant reentry could be defined in 94% of the patients. In two patients, a dominant pathway could not be delineated. In both cases, the cycle length was completely covered by recorded electrogram activity, and mid-diastolic fractionated electrograms were found around a tiny area with double potentials. They were therefore classified as micro-reentries. A critical isthmus could be identified in 89% of patients. All critical isthmuses were found in areas with a reduced conduction velocity and low voltage. Fractionated electrograms in these regions could be found, with an average duration of 150 ± 48 ms and voltage of 0.15 ± 0.14 mV. The shortest electrogram measured 81 ms, the longest was 234 ms, and the minimal voltage was 0.04 mV. Out of the 31 macro reentries, 7 (22%) were treated using a standard line (modified anterior line: *n* = 5; roof line: *n* = 2), 13 (42%) received an individual line, and 11 (36%) had a critical isthmus ablation. In three patients with perimitral flutter, a rather broad slow conducting region was found anterior of the left atrial appendage without the presence of double potentials or a dense scar. As this region was located at the site of a modified anterior line, this line was chosen as an ablation strategy. Only one AT was found to be related to a gap in the previous PV line. This AT presented with two gaps, marking the entry and exit through the PV area, and it was therefore classified as a small area.

### 3.3. Primary Endpoint

**Figure 3:**
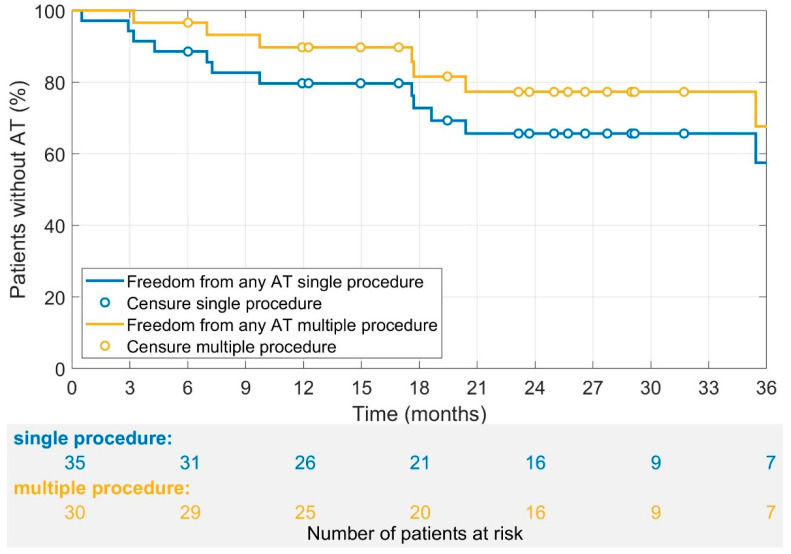
A Kaplan–Meier analysis for the freedom from any AT for multiple and single procedures per patient. The mean follow-up time was 30 ± 13 months for multiple procedures. Censored cases were indicated with yellow and blue circles. No blanking period was defined. The 12- and 24-month probability of freedom from any AT was 80% and 65% for single procedure and 90% and 77% for multiple procedures, respectively.

**Figure 4:**
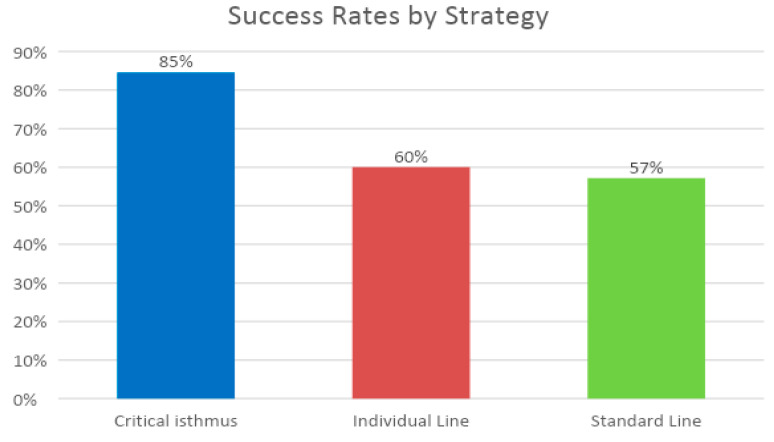
Clinical success dependent on the ablation strategy. Multi-procedure success after a mean follow-up time of 30 months. Differences were not significant. (Pearson Chi Square *p* = 0.28 and *p* = 0.12 when comparing critical isthmus vs. other lines).

The multi-procedure success (1.14 procedures per patient) for freedom from any AT at 12 months after the last procedure was 90. The single procedure success for freedom from any AT was 80%. After a mean follow-up time of 30 ± 13 months, the multi-procedure success rate (1.14 procedures per patient) for the freedom from any AT was 77%, and the single procedure success rate was 65% (Figure 3). The success rate of the critical isthmus approach was the highest, with 85%, followed by individual line (60%) and standard line (57%), as illustrated in Figure 4. 

### 3.4. Secondary Endpoints

#### 3.4.1. Termination into SR during CA

The first secondary endpoint, the termination into SR during CA, could be reached in all patients. The second secondary endpoint, the termination into a sinus rhythm upon the first ablation strategy, was reached in 32 (91%) of the patients. 

During radiofrequency (RF)-energy delivery, the conversion to other AT forms was observed in three patients. Those ATs were mapped and ablated as described above. Conversion to AF did not occur. Procedural data are displayed in Table 2. The total procedure time was 193 ± 57 min, the mean fluoroscopy time was 12 ± 8 min, the mean fluoroscopy dosage was 621 ± 555 cGycm^2^, and the mean total RF time was 24 ± 16 min. A peculiarity of this study was that a major effort was spent on a high, homogeneously distributed point density in all areas of the map and on a detailed post-analysis with manual annotation of the electrograms. The mean mapping time was 28 ± 10 min. 

#### 3.4.2. Time to Termination

**Figure 5:**
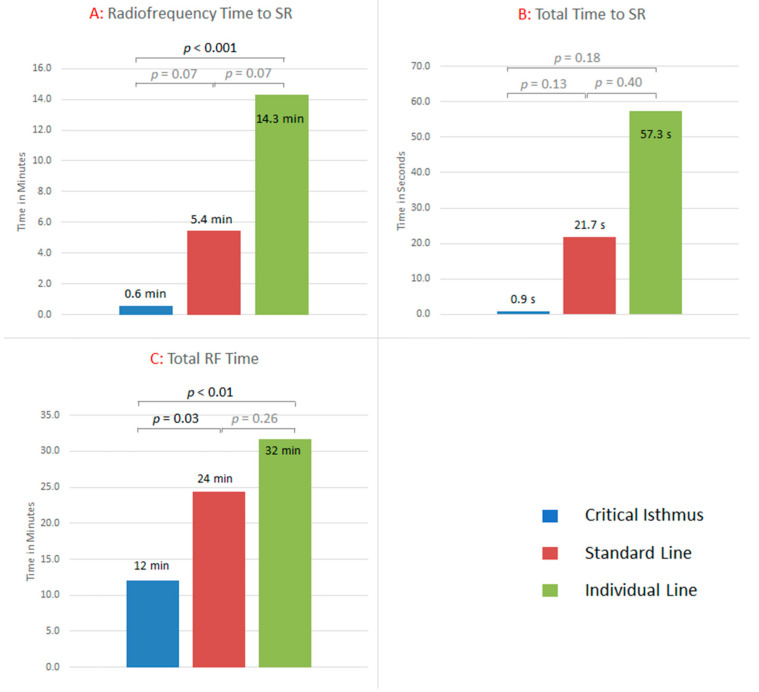
(**A**): RF time from the start of the first ablation until the termination in SR. For critical isthmus, individual line, and standard line, the RF time was 1 ± 1 min, 5 ± 9 min, and 14 ± 12 min, respectively. Standard lines took significantly longer compared to critical isthmus ablation (*p* < 0.001). (**B**): The total time to SR was 1 ± 1 min, 22 ± 49 min, and 57 ± 100 min, respectively. Differences were statistically not significant. (**C**): The total RF time, including the primary strategy and all additional ablations (i.e., achievement of bidirectional block, ablation of other critical isthmus, or fractionated potentials; re-PVI; and CTI ablation), was 12 ± 11 min, 24 ± 15 min, and 32 ± 12 min, respectively. Standard lines and individual lines took significantly longer than critical isthmus ablation. RF = Radiofrequency; SR = Sinus rhythm.

A total of 32 (91%) patients terminated in SR upon the first ablation strategy. In total, 100% terminated in SR during RF-delivery. The RF application time to restore SR differed between groups. It was significantly shorter in critical isthmus strategies (1 ± 1 min) compared to individual line (5 ± 9 min) and standard line (14 ± 12 min) (*p* < 0.01) strategies. The number of RF applications to restore SR was 1 ± 1, 6 ± 9, and 16 ± 13 for critical isthmus, individual line, and standard line, respectively. The total RF time was also significantly shorter in critical isthmus strategies (12 ± 11 min) compared to individual line (24 ± 16 min) and standard line (32 ± 12 min) approaches (*p* < 0.01) (see Figure 5). The total RF time included all additional ablations. The analysis of the total time to SR showed a trend towards shorter critical isthmus strategies (1 ± 1 min) compared to individual line (22 ± 49 min) and standard line approaches (57 ± 100 min) (*p* = 0.10).

#### 3.4.3. Non-Inducibility of Any Type of Arrhythmia

After restoring the sinus rhythm and completing the AT ablation, re-PVI was performed if indicated. PVs were reconnected in 20 patients (57%). A total of 2.4 PVs per patient presented PV potentials. All PVs could be successfully isolated by gap ablation on the previous ablation line, proven by entry block testing. In 7 (20%) patients, additional CTI ablation was performed. After the ablation, in 30 out of the 35 cases (86%), non-inducibility of any type of arrhythmia could be demonstrated (Table 3). The mean follow-up time was 30 ± 13 months. During the follow-up time, five patients needed a redo procedure due to the recurrence of AT. 

#### 3.4.4. Learning Curve

The learning curve analysis showed significant differences in the success rates and procedure time. Freedom from any AT was 20% for the first 5 procedures, as opposed to 80% for the last 5 procedures. Total procedure time was 251 ± 77 min and 149 ± 40 min (*p* = 0.04), respectively. 

## 4. Discussion

The demand for a more successful treatment of AT has been rising as a consequence of increasing numbers of cardiac surgery and catheter-based ablation of AF [37]. However, recurrent AT in pre-ablated atrial tissue is challenging to map and interpret, due to an incomplete understanding of the mechanisms as a consequence of areas of scar, low amplitude electrograms, and a multiplicity of arrhythmias within a given patient [38]. According to van Marion et al., the electro-pathological substrate remains poorly understood, and hence, defining the optimal approach per individual patient is challenging, whilst electro-pathology cannot be evaluated [39]. With the advent of high-resolution 3D mapping, some of these obstacles might be overcome by identifying conduction circuits in more granular detail [25]. However, even if the AT has been completely mapped using high resolution 3D mapping systems, the classification varies within the EP community. Differences have mainly been described concerning macro-reentry and focal tachycardias, leading to different incidences in the literature [17,18,40]. We studied 35 recurrent AT patients with the high-resolution 3D electro-anatomical mapping system Rhythmia HDx^TM^ in order to best confirm the hypothesized advantages of a standardized, algorithm-based ablation approach for complex arrhythmias resulting from potential benefits of complete mechanism traceability. Despite the availability of high-resolution 3D electro-anatomical mapping since several years ago, there is still no standardized terminology in the literature for whether macro-reentry tachycardias circle around a central obstacle of a certain size [24,41] or around an anatomical structure [42]. In this study, tachycardias circling around an anatomical structure were classified as macro-reentry. The term “focal” was used in some studies [40,41,42] for both micro-reentry and true focal automatisms, making it difficult to compare reported incidences and success rates. Non-reentrant focal tachycardias may have so far not been mapped accurately enough and have therefore been difficult to differentiate from micro-reentry tachycardias [43]. In our study, micro-reentries were separated from focal tachycardias by a manual analysis of local electrograms. We identified 86% macro- and 14% micro-reentry mechanisms. Focal automatisms or rotors could not be detected, with respect to the definition used in this study. Hence, with the new high-resolution 3D mapping systems, the focal or centrifugal origin hypothesis might be challenged for post-PVI patients. Decisive for our ablation approach was the determination of the dominant reentry and the number of critical isthmuses contained. Macro-reentries were only designated as such if they circled around an anatomical obstacle. Standard lines were only used in macro-reentries if no critical isthmus and no area of slow conduction could be found within the dominant reentry. Micro-reentry tachycardias were distinguished from focal automatisms by means of a manual signal analysis and were ablated accordingly. All other tachycardias were treated by an individual approach creating an ablation line between two non-conducting anatomical structures, containing the critical isthmus and slow conducting areas of the dominant reentry and passing through as little healthy tissue as possible. This strategy combines the tailored approach [24] and the critical isthmus ablation [25,26]. The primary intent of the study was to terminate the AT as fast as and with as little damage to healthy tissue as possible in order to achieve long-term benefits for the patients with left atrial tachycardia, following catheter ablation of atrial fibrillation. Non-inducibility was demonstrated after the ablation of all concomitant ATs. Derval et al. mainly used standard lines for ablation and reported 54% freedom from any AT after 12 months in 111 post-PVI patients in a comparative study [42]. The high percentage of a previous substrate modification and the proportion of macro-reentry tachycardias was comparable to our study population. The number of right atrial ablations was higher in the comparative dataset [42]. In our cohort, the success rate was 82% for single and 90% for multiple procedures using the individualized approach. Comparing results specifically for macro-reentry tachycardias, the ratio of anatomical to individual lines was 22%/78% in our data, as compared to 89%/11% in the comparative cohort. In our study, anatomical lines were only applied if the macro-reentry tachycardia showed no individual substrate or arrhythmia-specific regional conduction slowing. For peri-mitral ATs, we applied, in all cases, a modified anterior line, whereas the mitral isthmus line was predominantly used in the comparative study [42]. Takigawa et al. also introduced a tailored approach [24]. They defined the practical isthmus as the narrowest bridge of conducting tissue between scars or anatomical obstacles, which was often found to be different from the anatomical isthmus. ATs could be terminated in 74%, and the success rate after 6 months was 74%. Adapting and further developing this approach, Jungen et al. reported 73% freedom from AT after seven months and a mean of 1.4 procedures per patient [41].

In this study, the different ablation strategies were used according to the type of reentry and the number of critical isthmuses. The individual line combines the tailored approach [24] and critical isthmus ablation [25,26] in those patients with more than one critical isthmus. All ATs could be terminated into SR, and 91% terminated into SR upon the first ablation strategy. A bidirectional block was confirmed for all lines by stimulation. Freedom from AT could be demonstrated in 80% and 57% for single and 90% and 77% for multiple procedures after 12 and 30 months and a mean of 1.14 procedures, respectively. Patients receiving a critical isthmus ablation showed the best outcome. The average ablation time in this group to restore SR was 1 min, with a clinical success rate of 85% after a mean follow-up time of 30 months. The individual line was ranked second, while the anatomical line brought up the rear. However, the short-cut conclusion, that a critical isthmus ablation is superior to other strategies, was not valid, as the applied ablation strategy was chosen based on the underlying individual substrate. Rather the existing substrate was clearly defined and less complex in patients receiving a critical isthmus ablation. For patients who received a standard line due to a lack of areas with significantly reduced conduction velocity, the natural question arises of whether a pathological substrate might have not been recognized due to the current limitations of the available technologies. However, the acute and, in particular, 12-month and mean long-term success rates of this study reinforce the need of individual approaches in these complex patients. Furthermore, our study, to our knowledge, for the first time, covered a midterm follow-up of up to 41 months. While achieving similar acute results after 30 days like Schaeffer et al. [40], the newly applied specific individualized approach led to a success rate of 83% after one year, with relapses being associated with a higher age. We could also report a mid-term freedom from any AT rate of 65% for single and 77% for multiple procedures after a mean follow-up time of 30 months in these complex patients. The multi-procedure success rate showed only a slight improvement, which seemed unexpected but might be caused by the extremely diseased atria in these patients with multiple ablations and progressed substrate development.

### Limitations 

This was a single-center investigation, with a high selective patient cohort of low numbers. Only patients with ongoing stable AT and previous PVI were included in the study. It is therefore worth discussing if the findings and success rates could be transferred to a wider AT population. To overcome the limitations of the visualization of the 3D mapping system due to electrograms presenting with very low voltage, a manual electrogram analysis, to define the critical parts of the dominant reentry, was performed in this study. It is questionable if such an effort can be implemented in everyday clinical practice and whether inter-observer variabilities may lead to different results. Therefore, there is a demand for automatic algorithms to shorten, standardize, and simplify the analysis [44] and to prove the practicability of this approach in larger multi-center studies. The lowest electrogram amplitude recorded in a critical isthmus measured 0.015 mV. This demonstrates the need for a high-resolution catheter and raises the question of whether even an improved signal-to-noise ratio would add additional information to the understanding of the arrhythmia. Jungen et al. demonstrated high effectiveness and very low recurrence rates of CA in clinical tachycardia [41]. However, even if non-inducibility could be demonstrated at the end of the ablation, recurrencies of other arrhythmias were reported to be high. Therefore, an interesting question is whether computer simulation could help to identify further arrhythmia-specific substrates and, thus, could increase CA success rates [45].

## 5. Conclusions

With currently available ablation strategies, in combination with a predefined workflow based on the reentry mechanism and the number of critical isthmuses, a multiple procedure success rate of 90% for any AT recurrence at one-year could be reached. The more precisely the underlying substrate can be defined, the less ablation time is required. Tachycardia with a single critical isthmus can be ablated by critical isthmus ablation only, but the vast majority still require lines. 

## Figures and Tables

**Table 1:** Baseline characteristics.

Baseline Characteristics Index Procedure	Mean ± SD or Total (%) *n* = 35
Age (years)	65 ± 7
Gender, female (*n*)	12 (40%)
Hypertension (*n*)	26 (87%)
CAD (*n*)	8 (27%)
Diabetes mellitus (*n*)	3 (10%)
LVEF < 50%	7 (20%)
CHA2DS2-VASc	2 ±1
LA size (mm)	43 ± 6
LA area (cm^2^)	23 ± 5
**AAD**	
Betablocker (*n*)	21 (60%)
Amiodarone (*n*)	6 (17%)
Other (*n*)	0 (0%)
Number of prior left LA procedures (*n*)	2 ± 1
Time since last LA ablation (months)	18 ± 23
Prior LA-AT ablation (*n*)	13 (37%)
**Prior LA ablation approaches (*n*)**	
PVI only (*n*)	10 (29%)
PVI + CFAE(*n*)	5 (14%)
PVI + LA-Lines (*n*)	9 (26%)
PVI + CFAE + Lines (*n*)	8 (23%)
PVI + Rotor (*n*)	3 (9%)
Prior CTI ablation (*n*)	15 (43%)

CAD = coronary artery disease; LVEF = left ventricular ejection fraction; LA = left atrium; AAD = antiarrhythmic drugs; AT = atrial tachycardia; PVI = pulmonary vein isolation; CFAE = complex fractionated atrial electrograms; CTI = cavotricuspid isthmus. The AT was terminated immediately at the entry. The final ablation concept was the restoration of the completeness of the PV line with the closure of all gaps. The three micro-reentries were treated with a critical isthmus ablation, including the ablation of fractionated electrograms in the immediate vicinity.

**Table 2:** Procedural Data.

Procedural Data	*n* = 35
Termination in SR (*n*)	35 (100%)
Termination in SR upon 1st ablation strategy (*n*)	32 (91%)
Total RF time (min)	24 ± 16
Total procedure time (min)	193 ± 57
Total fluoro time (min)	12 ± 8
Total fluoro dosage (cGycm2)	621 ± 555
Non-inducibility (*n*)	30 (86%)
Follow-up time (months)	30 ± 13
Complications (*n*)	2 (6%)
Minor	2 (6%)
Major	0 (0%)
Ablation strategy (*n*)	
Standard line	7 (20%)
Individual line	15 (43%)
Critical isthmus	13 (37%)
Focal	0 (0%)
Local area	0 (0%)
Total time to SR (min)	21 ± 57
Standard line	57 ± 100
Individual line	22 ± 49
Critical isthmus	1 ± 1
RF time to SR (min)	5 ± 9
Standard line	14 ± 12
Individual line	5 ± 9
Critical isthmus	1 ± 1
Total RF applications to SR (*n*)	6 ± 9
Standard line	16 ± 13
Individual line	6 ± 9
Critical isthmus	1 ± 1

“Total time to SR” = Begin RF Ablation to SR; “Total RF time” includes Re-PVI and CTI. Complications: 1× pericardial effusion and 1× groin hematoma. Both were treated conservatively and did not receive additional interventions.

**Table 3:** Mapping Data.

Mapping Data	*n* = 35
AT cycle length (ms)	297 ± 86
P-wave duration in AT (ms)	117 ± 32
ATs mapped (*n*)/patient	1 ± 1
Points per map (*n*)	20,773 ± 9748
LA volume (mL)	188 ± 48
Mapping time (min)	27 ± 10
Dominant reentry defined (*n*)	33 (94%)
Critical isthmus present (*n*)	31 (89%)
Type of AT (*n*)	
Macro	31 (88%)
Small area	1 (3%)
Micro	3 (9%)
Focal	0 (0.0 %)
Rotor	0 (0.0 %)
PV-reconnection (*n*)	47 (33 %)
PV-reconnection per patient (*n*)	1.6 ± 1

AT = atrial tachycardia; LA = left atrium; WOI = windows of interest; PV = pulmonary vein.
